# Diagnosis, care pathways, and Complementary and Alternative Medicine (CAM) use among digestive cancer patients in Benin: a qualitative study

**DOI:** 10.1007/s00520-026-10853-1

**Published:** 2026-06-09

**Authors:** Tassadit Merabtine, Maxime Adjignon Sognigbe, Elodie Marcellaud, Jean Toniolo, Zeinab Tarhini, Habib Ganfon, Aboudou Raïmi Kpossou, Niki Christou, Dieu donné Gnonlonfoun, Jeremy Jost

**Affiliations:** 1 Inserm U1094, IRD UMR270, Univ. Limoges, CHU Limoges, EpiMaCT - Epidemiology of Chronic Diseases in Tropical Zone, Institute of Epidemiology and Global Health – Michel Dumas, Omega Health, Limoges, France; 2 Laboratory of Chronic and Neurological Diseases (LEMACEN), Faculty of Health Sciences, University of Abomey-Calavi, Cotonou, Benin; 3 Faculty of Health Sciences, University of Abomey-Calavi, Cotonou, Benin; 4 Clinical Pharmacy Unit, Pharmacy Department, University Hospital of Limoges, Avenue Martin Luther King, 87000 Limoges, France; 5 University Department of Nursing Science, Faculty of Medicine, Univ. Limoges, Limoges, France; 6 Hubert KM National Hospital and University Center (CNHU-HKM), Cotonou, Benin; 7 Laboratory INSERM U1308, CAPTuR, Control of Cell Activation in Tumor Progression and Therapeutic Resistance, Medical School– 2 Rue du Docteur Marcland, 87025 Cedex Limoges, France; 8 Digestive Surgery Department, University Hospital of Limoges, Avenue Martin Luther King, 87000 Limoges, France; 9 Department On Neurology, Hubert KM National Hospital and University Center (CNHU-HKM), Cotonou, Benin

**Keywords:** Digestive cancers, Complementary and alternative medicine, Diagnostic delay, Care pathways, Benin

## Abstract

**Purpose:**

In Benin, patients with digestive cancers face multiple challenges, including diagnostic delays and limited access to care. In this context, the use of complementary and alternative medicine (CAM) is common. This study aimed to describe the diagnostic and care pathways of patients with digestive cancers in Benin, and their experiences with both complementary and alternative medicine (CAM) and conventional treatments.

**Methods:**

This qualitative study was conducted at the University Hospital Center (CNHU-HKM) in Cotonou, Benin, among adult patients diagnosed with primary digestive cancers who reported CAM use. The sample was determined by data saturation and included 11 patients. Semi-structured, face-to-face interviews were conducted, audio-recorded, and fully transcribed. A thematic analysis was performed followed by investigator triangulation.

**Results:**

Patients frequently reported diagnostic delays linked to initial symptom minimization, medical wandering, and low awareness of digestive cancers. Access to care was further constrained by financial difficulties and limited availability of medicines. The use of complementary and alternative medicine was mainly motivated by financial barriers, dissatisfaction with conventional treatments, management of side effects, and the search for well-being. Finally, patients emphasized the need for holistic care, including psychosocial and nutritional support, and physician involvement in CAM supervision.

**Conclusion:**

This study underscores the complex trajectories of digestive cancer patients in Benin, marked by diagnostic delays, inequities in access to care, and the central role of CAM. The findings call for holistic, patient-centered models of care that promote early detection, enhance access to services, and ensure the safe and culturally sensitive integration of CAM.

**Supplementary Information:**

The online version contains supplementary material available at 10.1007/s00520-026-10853-1.

## Introduction

Cancer remains one of the leading non-communicable diseases worldwide and represents a major public health challenge. In 2022, an estimated 20 million new cases and 9.7 million cancer-related deaths were reported globally [1, 2]. Gastrointestinal (GI) cancers account for nearly one-quarter of all cancer cases and one-third of cancer-related deaths [3].

In sub-Saharan Africa (SSA), these cancers continue to receive limited attention in health agendas, despite projections indicating a 73% increase in incidence by 2030, significantly higher than the global average. Most cases are diagnosed at advanced or metastatic stages, particularly in low- and middle-income countries where diagnostic capacity and treatment options remain limited [4]. Although early detection is associated with improved survival, nearly half of cancers are identified at advanced stages [5].

In Benin, a recent study reported an average delay of nine months between symptom onset and the first medical consultation, with an average overall survival of ten months [6]. Contributing factors to late diagnosis may include limited public awareness of symptoms, prevailing cultural beliefs, initial reliance on non-conventional treatments, as well as health system challenges such as misdiagnosis, understaffed facilities, weak referral pathways, and inefficient scheduling [7].

Complementary and Alternative Medicine (CAM) refers to health practices and products not typically included in conventional medical care. According to the National Cancer Institute, CAM can be grouped into five categories: mind–body practices (e.g., meditation, yoga, tai chi), biologically based practices (e.g., vitamins, dietary supplements), manipulative and body-based practices (e.g., massage therapy, chiropractic therapy), energy healing (e.g., reiki), and whole medical systems (e.g., Ayurvedic medicine, Traditional Chinese Medicine, naturopathic medicine) [8].

In many Western frameworks, complementary and alternative medicine (CAM) is defined in opposition to conventional biomedical care. However, this distinction does not fully reflect the realities of healthcare systems in Benin and other West African countries. Practices such as herbal medicines, prayer, and other spiritual approaches often categorized as CAM in the literature are deeply rooted in cultural norms and are widely used as part of routine health-seeking behaviors. In this context, what is labeled 'alternative' from a Western biomedical perspective may, in fact represent a primary or mainstream therapeutic pathway. We use the term CAM for consistency with the existing literature, while acknowledging that this framing does not fully capture the cultural legitimacy of these practices in the local context.

In Africa, the use of these practices, products, and approaches among cancer patients is highly prevalent, with reported rates ranging from 36 to 80% across countries [9]. Commonly reported practices include herbal remedies, spiritual healing, massage, dietary supplements, and various nutritional interventions [9]. Patients’ motivations are shaped by cultural beliefs, perceptions of greater accessibility and affordability compared with standard therapies, the desire to relieve symptoms or treatment-related side effects, hopes for psychological and spiritual well-being, and the wish to regain a sense of control over their illness [10]. However, the concurrent use of CAM and conventional therapies carries potential risks, including delayed initiation of biomedical treatment, harmful drug interactions, adverse effects, and a possible reduction in the effectiveness of conventional therapies [11, 12]. In addition, disclosure rates remain low, largely due to patients’ fear of disapproval or the lack of proactive inquiry from providers [13, 14]. Despite these risks, some patients report perceived benefits such as improved quality of life, pain relief, and enhanced emotional well-being [15].

Although late diagnosis of digestive cancers and recourse to CAM are widespread in Africa, there is a lack of qualitative studies describing diagnostic trajectories, care pathways, and the use of CAM among patients with digestive cancers, as well as how these patients integrate or alternate between conventional and non-conventional treatments. In Benin, in particular, such qualitative data are currently lacking.

This qualitative study aimed to explore the diagnostic processes and care pathways of patients with digestive cancers in Benin, highlighting the obstacles they face and their experiences with both complementary and alternative medicine and conventional treatments.

## Methodology

### Study design

This cross-sectional, exploratory, monocentric qualitative study was conducted in accordance with the Consolidated Criteria for Reporting Qualitative Research (COREQ) guidelines [16].

### Study setting and population

The study was conducted at the Hubert Koutoukou Maga National University Hospital Center (CNHU-HKM) in Cotonou, Benin. Eligible participants were adults (18 years and older) with a confirmed diagnosis of primary digestive cancer, who reported the use of complementary and alternative medicine and provided informed consent. Patients were identified through outpatient and inpatient registers as well as medical records from the departments of Visceral Surgery, Internal Medicine and Palliative Care, and Gastroenterology, between January 2023 and January 2025. Recruitment was carried out from May to July 2025.

### Participant recruitment

Patients identified from registers were first contacted by phone calls and screened for CAM use with a brief questionnaire listing various types of complementary and alternative medicine. During the call, the concept of CAM was explained to ensure a shared understanding. For this study, CAM was defined as health practices and products not typically included in conventional medical care, encompassing mind–body approaches (e.g., meditation, yoga), biologically based products (e.g., dietary supplements, medicinal plants), manipulative techniques (e.g., massage, chiropractic care), energy-based therapies (e.g., reiki), and whole medical systems (e.g., Traditional Chinese Medicine, Ayurveda, naturopathy) [8]. Patients were considered CAM users if they reported any form of use whether active or passive and even a single attempt was sufficient for classification. Those identified as CAM users were invited to participate in the study, and consenting individuals were scheduled for individual face-to-face interviews.

### Sampling strategy

A purposive, non-probability sampling strategy was employed. The sample size was determined progressively during data collection, following the principle of thematic saturation. Based on the literature, at least nine interviews were expected to be required to reach saturation [17]. Saturation was considered to have been reached when no new themes or insights emerged from the interviews [18]. To confirm that there are no new themes emerging, two additional interviews were conducted after saturation was achieved.

### Data collection

The research team comprised researchers with backgrounds in public health and pharmacy, including both local (Beninese) and non-local members. Semi-structured, face-to-face interviews were conducted by two researchers: one PhD candidate in public health (TM) and one final-year pharmacy student from Benin (MAS), both trained in qualitative research methods under the supervision of a senior researcher (JT) who is a nurse and holds a PhD in public health, with expertise in qualitative and mixed-methods research, particularly in oncology.

The interviews were conducted in the local language Fongbé and in French, depending on participants’ preference. All interviews were audio-recorded with participants’ written informed consent and supplemented by field notes. Anonymous transcriptions were first translated into French, and then into English.

### Interview guide

The interview guide was developed based on a review of the literature and pre-tested on one patient, who was not included in the final data analysis [19]. Following the pre-test, modifications were made to the wording of a question and the order of items to improve clarity and flow. It began with an introductory framework presenting the research team and the study objectives. The guide comprised eight main questions with potential follow-up prompts. The opening question was deliberately broad to minimize interviewer influence.

The interviews explored the circumstances and modalities of diagnosis, the barriers to accessing diagnostic tests and care, patients’ experiences with conventional and non-conventional treatments, types of CAM used, motivations for CAM use, perceived outcomes, as well as expectations regarding their well-being and the integration of CAM. Examples of guiding questions included:

- “Could you tell us your story with cancer, from the first symptoms to the announcement of the diagnosis?”
- “Did you face any difficulties in accessing diagnostic tests? If yes, which ones?”
- “In your opinion, what could help improve the early diagnosis of the disease?”
- “How did you experience your medical management with conventional treatments (chemotherapy, radiotherapy, surgery, stoma, etc.)?”
- “What types of complementary or alternative medicine have you used?”
- “What motivated you to turn to these practices?”
- “What kinds of changes, if any, did you notice after using these complementary medicines or therapies?”
- “What do you expect from healthcare professionals regarding your well-being and the integration of complementary medicine into your care?”

Each interview was concluded by thanking patients for their valuable participation and sharing of experiences, and by asking whether there was anything else they wished to add that had not been covered during the interview. At the end of the guide, a section was included to collect socio-demographic information such as age, sex, educational level, and socio-economic status.

The interview guide used during face-to-face interviews with patients is presented in (Supplementary Material 1).

### Data analysis

The interview recordings were fully transcribed verbatim by the first and the second author. Data analysis began with repeated readings of the transcripts and field notes to identify general ideas and note our first impressions [20]. An analytic journal was kept throughout this stage to document preliminary reflections and emerging insights.

Data were then coded using NVivo 15® by three authors, examining transcripts line by line to identify specific meaning units. An inductive approach was employed, allowing codes to emerge directly from the data, after which themes were interpreted in relation to relevant theoretical frameworks to enhance analytical depth. All codes were documented with verbatim examples. At the same time, the analytic journal was used to track the evolution of reflections and emerging ideas [21].

Once codes were generated, they were grouped into themes and sub-themes in accordance with Braun and Clarke’s guidelines for thematic analysis in qualitative research [22].

Researcher triangulation was carried out, and each theme was critically examined to ensure it faithfully reflected the raw data. A collaborative review among the researchers helped refine interpretations and ensure data triangulation, with adjustments made to eliminate inconsistencies and overlaps [23]. An in-depth analysis of each theme was then conducted to clarify its meaning and role in addressing the research question. Theme names were chosen to be simple, clear, and representative.

The socio-demographic and clinical characteristics of the included patients were summarized using percentages for categorical variables and means ± standard deviations for continuous variables. Normality of continuous variables was assessed using the Shapiro–Wilk test in JASP software.

### Researcher reflexivity and bias mitigation

Several measures were implemented to ensure rigor and minimize potential bias. The interview guide was pre-tested, and questions were refined to improve clarity and avoid misunderstandings. In addition, a role-play session was conducted with pharmacy colleagues in Benin to ensure that the questions accurately captured the intended meaning and were culturally appropriate. Field notes were taken independently by both interviewers during each interview, and audio-recorded interviews were transcribed collaboratively to ensure consistency between the recordings and the notes. Each interviewer maintained an analytic journal throughout the research process to document emerging ideas and reflexive considerations. Data analysis was conducted independently by three researchers, followed by a triangulation process during which interpretations were compared and discussed to ensure consistency and reduce bias.

The researchers’ backgrounds in public health and clinical disciplines may have influenced both data collection and interpretation, particularly by shaping the framing of questions and the attention given to biomedical aspects of care. The presence of both local and non-local researchers may also have affected participants’ responses, potentially encouraging socially desirable answers or influencing how complementary and alternative medicine practices were described. These positional dynamics were actively considered during data analysis and interpretation through reflexive discussions among the research team.

### Ethical considerations

The study received approval from the Health Sciences Research Ethics Committee (CER-SS) of the University of Abomey-Calavi, Benin (DECISION N° 010–2025/CER-SS). Data collection authorization was granted by Hubert KM National Hospital and University Center (CNHU-HKM), Cotonou, Benin, and all patients provided written informed consent.

## Results

**Fig. 1:**
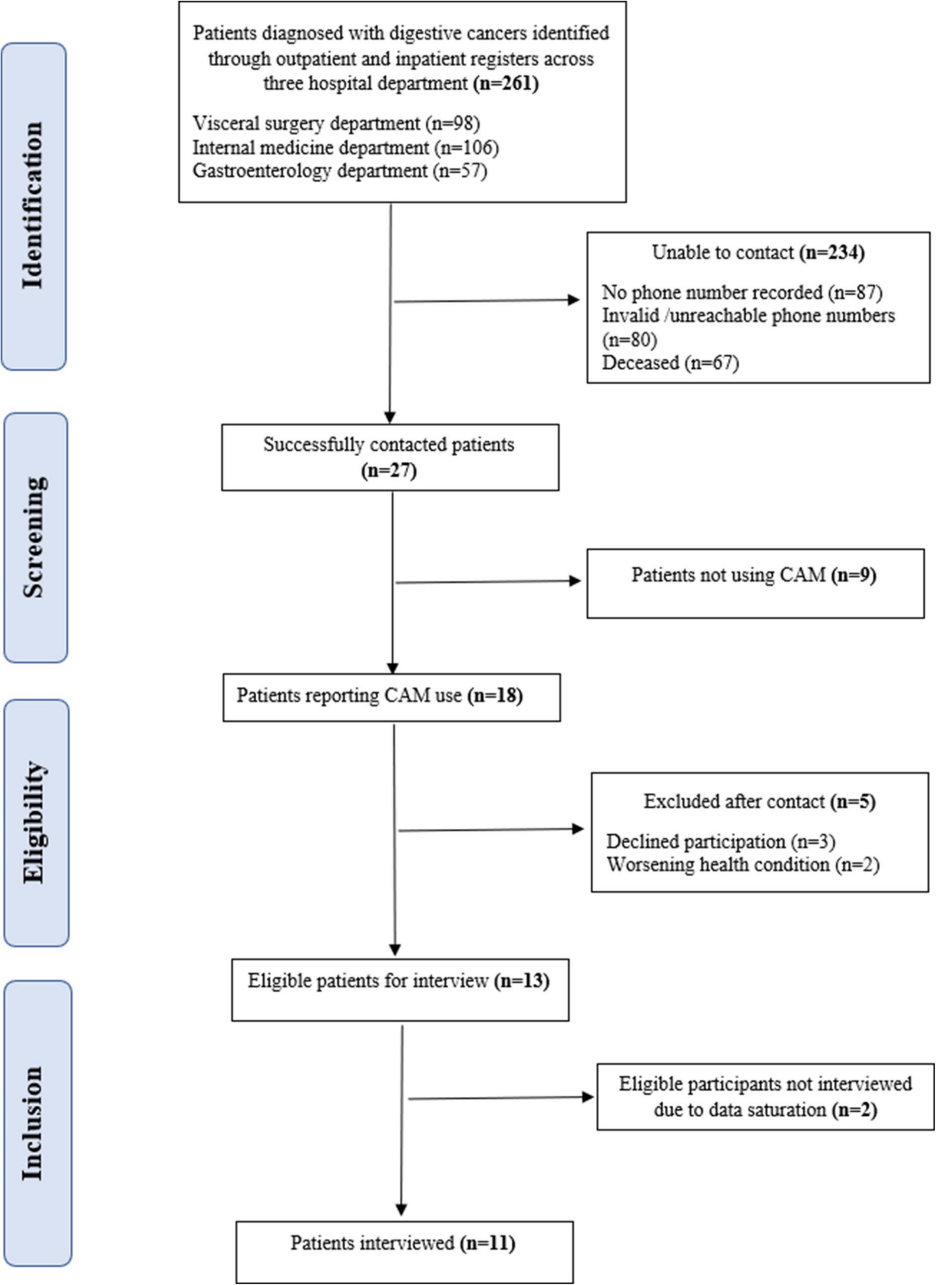
Flow diagram of patient’s identification, screening, eligibility, and inclusion

A total of 261 patients diagnosed with digestive cancers were identified across various hospital departments (visceral surgery: 98; internal medicine:106; gastroenterology: 57). Among them, 87 had no phone number recorded, 80 had unreachable numbers, and 67 were deceased, leaving 27 patients successfully contacted. Of these, 9 were non-users and 18 were users of complementary and alternative medicine. Among the 18 CAM users, 11 agreed to participate and were interviewed. The remaining seven did not participate in the study: three declined to take part, two were unable to participate due to a worsening health condition, and two eligible participants were not interviewed because data saturation had already been reached. Thematic saturation was achieved after the ninth interview; however, two additional interviews were conducted for confirmation, resulting in a final sample of 11 participants (Fig. 1). The average interview duration was 38.1 ± 17.4 min.

All patients had a diagnosis of digestive cancer, most frequently colorectal cancer 63.6% (*n* = 7), followed by pancreatic cancer 18.2% (*n* = 2), while gastric and liver cancers each accounted for 9.1% (*n* = 1) of the sample. Among the 11 patients, the majority were male 81.8%, with a mean age of 51.6 ± 12.4 (min = 33, max = 70). All participants were married or cohabiting with a partner. Nearly half (45.5%) were retired, and more than half (54.5%) had completed a university education.

The Table 1 bellow summarizes the socio-demographic and clinical characteristics of the patients interviewed.

**Table 1:** Socio-demographic and clinical characteristics of the patients interviewed

Patient ID^i^	Sex	Marital status	Education level	Employment status	Age at diagnosis	Type of digestive cancer
P01	Male	Single	University	Unemployed	45	Colorectal cancer
P02	Male	Married	University	Employed	45	Colorectal cancer
P03	Male	Married	University	Retired	67	Colorectal cancer
P04	Male	Married	University	Retired	66	Pancreatic cancer
P05	Male	Married	Secondary	Retired	58	Pancreatic cancer
P06	Female	Married	Secondary	Unemployed	39	Stomach cancer
P07	Female	Married	Primary	Retired	56	Colorectal cancer
P08	Male	Married	University	Employed	33	Colorectal cancer
P09	Male	Married	University	Employed	45	Colorectal cancer
P10	Male	Single	Secondary	Employed	44	Colorectal cancer
P11	Male	Married	Secondary	Retired	70	Liver cancer

^i^Patient ID: From P01 to P11, these are identifiers assigned to patients according to their order of participation in the study

### Themes and sub-themes

Thematic analysis and triangulation resulted in the identification of six major themes and twenty-six sub-themes. These themes covered the processes of early symptom appraisal and misinterpretation, as well as the pathways and challenges involved in navigating cancer diagnosis within constrained health systems. They also addressed structural vulnerabilities affecting access to care, including financial, organizational, and insurance-related barriers.

Other themes explored the lived experience of cancer as a biographical disruption, including its physical, psychosocial, and financial impacts, as well as patients’ coping strategies and sources of support. In addition, the themes explored therapeutic navigation and medical pluralism, including patient’s experiences with conventional treatments as well as different profiles of engagement with CAM. They also addressed the types of CAM used, patients’ motivations, sources of information, perceived benefits, and the cultural meanings and legitimacy associated with these practices. Finally, the analysis highlighted patients’ expectations regarding holistic and integrated care, including the need for psychosocial support, medical guidance, and stronger involvement of the healthcare system. All themes and corresponding sub-themes, along with the number of citations, text passages, and illustrative quotations, are presented in (Supplementary Material 2).

#### Early symptom appraisal and misinterpretation

This theme captures how patients initially perceived and interpreted their symptoms prior to seeking medical care. Many initially minimized or trivialized their symptoms, attributing them to benign conditions. As one patient explained: *“…I thought it was just some passing stomach aches…I told myself, well, it’s not really a problem as such” (P01)*.

Testimonies also revealed a widespread lack of awareness regarding digestive cancers: *“…so here is cancer, which wasn’t something we used to hear much about in Africa. We were more familiar with diseases like malaria, hemorrhoids…. Now cancer comes along, like hemorrhoids which make it difficult to pass stool. So, if there could be an awareness campaign so that people are informed and know the measures or precautions to take to avoid it, that would help” (P07).*

#### Navigating diagnosis within constrained health systems

For several patients, a definitive diagnosis was only established once symptoms became severe or in an emergency context, as reflected in the following account: *“The constipation was there, and then it became more severe and intense, and even passing stool became difficult. That’s what made me go for a consultation quickly”*. Such delays were perceived as particularly burdensome: *“If the illness could be diagnosed earlier, I wouldn’t have had to suffer so much and I wouldn’t have had to spend so much”* (P05). This tendency to delay medical consultation was compounded by diagnostic wandering, with symptoms misattributed to common illnesses such as Malaria or bacterial infections*: “…then they said there’s the beginning of malaria or the beginning of a bacterial infection, that’s all, it’s nothing serious” (P03).*

Organizational barriers within health facilities created additional obstacles, including staff shortages and long waiting times to obtain an appointment: *“I wanted to have a colonoscopy in January, and they told me all the doctors were already booked, so my appointment was pushed to March” (P10)*.

Furthermore, some diagnostic examinations were sometimes described as distressing experiences, such as fibroscopy, referred to as *“disgusting” (P03).* Reactions to the disclosure of cancer varied widely, ranging from emotional pain *“At that moment, he told me it was stomach cancer, it hurt me a lot”, (P06)* to relative detachment *“They told me it was cancer and all that, I said it’s not a problem, you know..I wasn’t traumatized by the announcement, no, not at all”(P03*).

#### Structural vulnerability shaping access to care

Access to healthcare was restricted by both financial and organizational barriers. Several participants reported being unable to afford diagnostic tests or to complete their treatment: *“I encountered a lot of difficulties in accessing diagnostic tests, even the treatment I haven’t completed because I no longer have the means” (P06),“I had too much debt, so finding money to continue with chemotherapy and other treatments had become impossible, impossible, impossible”(P08).*

Beyond financial constraints, patients also reported challenges related to the availability and accessibility of medicines and healthcare services. Some prescribed drugs were simply unavailable, requiring patients to search extensively: *“Not only are these medicines out of stock, but you also have to search far to find them” (P01)*.

For employees benefiting from health insurance, its limitations were also highlighted: *“The medicines are not covered by insurance. If I want to do tests, it’s covered; for hospitalization, it’s covered; for procedures, it’s covered. But not for medicines, which are expensive (P06)”.*

#### Living with cancer: biographical disruption and adaptation process

Living with cancer was described as a profoundly disruptive experience, covering both physical and emotional suffering. Patients reported pain combined with psychological distress: *“There is also physical pain and emotional pain” (P05).* The psychosocial impact was significant, with mentions of mental exhaustion and strain on family life*: “…it ruins you mentally” (P02)*; *“It negatively affected the proper functioning of my family, whether it was the children who constantly saw everything I was going through… but also on the financial level” (P09).*

Despite these challenges, patients also described strategies of resilience and adaptation. Some emphasized the importance of inner strength and mental resilience: *“As for me, I am strong mentally I am strong, because the limits we set for ourselves are the limits of our mentality If you want to, you can” (P02)*. *“…in my life, I expect anything, because I know there is a solution for everything…. I am in the momentum of overcoming it” (P03).* Family emerged as an important source of support: *“If my parents had not supported me, I am not sure I would have been able to continue until the end” (P02).*

#### Therapeutic navigation and medical pluralism

Perceptions of chemotherapy varied. Some expressed satisfaction: *“The chemotherapy treatment is really beneficial” (P04)*, while others reported ineffectiveness: *“…The treatment with chemotherapy was not a success, it was a total failure” (P09)*. The intensity of chemotherapy sessions and their side effects were described as major obstacles. Commonly reported side effects included loss of appetite, throat dryness, skin affections, and overwhelming fatigue: *“For me, chemotherapy was difficult, very difficult. At the beginning, I had a loss of appetite…I had a dry throat… This whole thing really tires me out” (P05)*. In addition, the presence of a stoma was described as particularly burdensome and associated with discomfort: *“…ah, the stoma, wow, the stoma! waa, that was the most unpleasant thing” (P03).*

Patients demonstrated diverse attitudes towards traditional and CAM. Some expressed strong adherence (pro-CAM patients), considering CAM their primary treatment pathway, while viewing conventional medicine as secondary: *“It didn’t just bring me peace of mind, I would say it contributed 70% to my healing” (P02).* Others were more skeptical, using CAM cautiously while, prioritizing biomedicine (P01). Several adopted a hybrid approach, combining conventional and traditional medicine in search of balance: *“Because with conventional medicine and traditional medicine, we can try to find the right balance..” (P09).* Others were more passive, turning to traditional and CAM mainly due to recommendations from others or financial constraints: *“..It’s the cost that is quite high, the scan is 160,000 FCFA, and I spend at least 260,000 FCFA per treatment cycle. So, I turned to prayer and herbal teas” (P06).*

Reported practices included herbal medicine, aromatherapy, fasting, dietary supplements, cold baths, Chinese medicine, and consultations with traditional healers. Spirituality both religious and personal, also played a central role: *“Afterwards, I entrusted myself to God, and I noticed that things improved” (P04).*

Motivations for traditional and CAM use ranged from financial difficulties and dissatisfaction with conventional treatments to worsening symptoms or the search for improved quality of life. Patients’ main sources of information were internet searches and advice from their social circle. Some actively sought testimonials online (P02), while others turned to family, friends, or social media (P07). Overall, CAM was perceived as a tool that facilitated adaptation and improved quality of life: *“…So all of that really helped me to live better with the illness” (P05).*

#### Need for CAM recognition and holistic care

Patients highlighted the widespread use of CAM and the importance of supporting research in this field: *“This medicine needs to be supported, we need to engage in that research” (P02)*. They also valued an integrative approach combining conventional and traditional practices: *“Because with conventional medicine and traditional medicine, we can try to find a middle ground, to find some alignment that can still relieve patients” (P09).*

Beyond biomedical treatment, patients emphasized the need for psychological and psychosocial support: *“Psychological support would also be good” (P07).* Requests for dietary advice (P04) and physician involvement in the safe integration of CAM were also mentioned: *“If doctors also supported us regarding what we take or do outside of the care we receive at the hospital, that would be good…” (P04)*. Finally, establishing a trusting relationship with physicians was considered essential: *“…the way he explains things to you, gives you confidence, and that alone can heal you” (P10).*

## Discussion

The analysis highlighted the circumstances and pathways of cancer diagnosis, the barriers to accessing diagnostic tests and care, and patients’ experiences of illness, its trajectory, and the adaptation process, as well as their experiences with both conventional and non-conventional treatments.

Early diagnosis is a cornerstone of cancer control because of its strong association with survival [24]. However, in our study, the diagnostic process was often described as long, confusing, and delayed. Our findings align with evidence from the literature indicating that such delays often occur when patients fail to recognize and act upon suspicious symptoms. Low public awareness of early warning signs particularly when symptoms are atypical has been identified as a major reason for late presentation [25]. Additional barriers, such as lack of time to schedule a medical appointment, fear of what the doctor might discover, or discomfort in discussing symptoms, may also contribute to delays [26]. At the primary care level, challenges in symptom recognition, the lack of clear referral guidelines, and limited availability of specialized resources further exacerbate the delay [27].

Patients also expressed discomfort with some diagnostic examinations, such as colonoscopy, which were perceived as invasive and unpleasant. This aligns with qualitative research indicating that nearly half of patients report anxiety or emotional distress related to colonoscopy [28].

These findings underline the urgent need to strengthen early detection strategies, improve public awareness of digestive cancers, and promote sensitive communication between healthcare providers and patients.

Similarly, a study conducted in Tanzania by *Makene *et al*.* reported long diagnostic delays among cancer patients, with a median time of almost one year between first symptoms and diagnosis. These delays were strongly patterned by socioeconomic disadvantage and were linked to slow symptom recognition, limited diagnostic capacity, weak referral pathways, and difficulties paying for diagnostic tests. This supports our findings that delayed diagnosis in sub-Saharan Africa reflects not only patient-level factors, but also structural and health system barriers [29].

Access to care was further constrained by financial and organizational barriers. The inability to afford diagnostic tests or complete chemotherapy cycles was a recurrent theme, with some patients reporting heavy debts or abandonment of treatment. Such challenges are common in contexts where universal health coverage is limited and out-of-pocket expenses remain the main source of healthcare financing [30, 31].

Patients also reported experiencing shortages of essential medications and difficulties in obtaining them, compounded by long waiting times and inadequate staffing in health facilities. Similar barriers have been widely documented across many low- and middle-income countries (LMICs) and in global health reports, which underscore the structural inequities faced by cancer patients in resource-limited settings [32, 33]. These findings highlight the need for policies that enhance equitable, and effective cancer care.

The lived experience of cancer was described as profoundly disruptive, encompassing physical pain, emotional suffering, financial difficulties, and disruptions to family life. However, the accounts also revealed notable resilience and adaptation strategies. Receiving a cancer diagnosis is a stressful experience, often accompanied by a range of negative emotions. In this context, coping understood as the cognitive and behavioral efforts individuals employ to manage or adapt to stressful situations is often adopted by cancer patients. In this study, some patients described relying on their inner strength and determination to face the disease, while others emphasized the crucial role of family support in sustaining treatment adherence and coping with daily challenges [34, 35]. Spirituality also emerged as a central coping mechanism. Patients frequently reported that faith, prayer, and a sense of connection to a higher power provided comfort and hope, helping them to endure both physical and emotional suffering. For many, spirituality was not only a source of inner strength but also a way to interpret the meaning of their illness, reduce fear, and maintain resilience during treatment. Belief in divine intervention and the healing power of spiritual practices further underscores the importance attributed to faith in navigating the psychological challenges of cancer. Consistent with the literature, spirituality has been widely recognized as a key coping strategy among cancer patients, positively contributing to psychological adjustment and quality of life [36, 37].

Perceptions of conventional treatment were ambivalent. Chemotherapy was sometimes considered effective and beneficial, but at other times described as ineffective, burdensome, and associated with severe side effects such as fatigue, loss of appetite, and skin affections [38]. The presence of a stoma was particularly distressing for some patients, negatively affecting their quality of life and self-image. These findings are in line with the literature showing that patients living with a stoma commonly experience profound physical discomfort, social isolation, disturbances in body image, sexual dysfunction, and psychological distress [39]. Such experiences point to the need for supportive interventions that manage side effects, improve comfort, and provide psychological support throughout treatment.

Besides conventional treatments, patients also turned to complementary and alternative medicine, which they approached in diverse ways. Some patients strongly adhered to CAM, considering it central to their healing process, while others adopted hybrid strategies that combined biomedical care with CAM practices. For certain patients, the decision to use CAM was influenced by the advice of relatives or driven by the financial inaccessibility of conventional medicine. Reported practices included herbal medicine, aromatherapy, fasting, dietary supplements, cold baths, Chinese medicine, and consultations with traditional healers, while spirituality, encompassing both religious faith and personal beliefs, was deeply embedded in the illness experience. These practices were motivated by multiple factors, including financial barriers to conventional treatment, dissatisfaction with chemotherapy, worsening of symptoms, and the search for better quality of life. Mao et al. reported that the use of CAM is particularly common in low- and middle-income settings, often in response to restricted access to biomedical care and the heavy burden of out-of-pocket expenditures, which drive many patients toward options that are more financially accessible or culturally acceptable [40].

These findings can be interpreted through the lens of medical pluralism, which describes the coexistence and interaction of multiple therapeutic systems within a given context [41]. In this perspective, patients do not simply choose between conventional and alternative medicine but actively navigate and combine them in response to their needs, beliefs, and constraints. In line with this, integrative oncology frameworks highlight the growing need to consider complementary practices alongside biomedical care to improve patient-centered outcomes [40]

Evidence from Tanzania suggests that the use of traditional healing is not only driven by cultural beliefs, but also by challenges within the health system. Patients often turned to traditional healers after repeated unsuccessful consultations, worsening symptoms, or difficulties accessing diagnostic services [29].

Similar patterns have been reported in other sub-Saharan African contexts. In Ghana, qualitative studies have shown that many cancer patients initially rely on herbal medicine and spiritual healing before presenting to hospitals, often influenced by cultural beliefs and financial barriers. As in our study, these practices were frequently associated with disappointment, perceived inefficacy, and delays in accessing conventional care [42].

These findings should be interpreted within the broader context of medical pluralism in West Africa. In Nigeria, traditional medical practices have been described as mainstream ways of treating disease, with some individuals even viewing Western biomedicine as an alternative or complement to traditional medicine [43]. Similarly, in Benin, herbal medicine, spiritual healing, and consultations with traditional healers are not necessarily perceived as 'alternative' practices, but rather as culturally embedded components of patients' care pathways. This raises important questions about the transferability of Western CAM frameworks to sub-Saharan African settings, and underscores the need for culturally sensitive conceptual tools when studying therapeutic pluralism in low-resource contexts.

Our findings also reflect patients’ expectations for more holistic models of care, with a need for greater recognition of CAM and further research in this field. Several studies in oncology show growing interest in integrative approaches, where evidence-based complementary practices are combined with conventional treatment to improve symptom control, enhance quality of life, and respond to patients’ cultural values and beliefs [40, 44, 45]. In addition, patients expressed the importance of dietary advice, psychological support, and stronger physician involvement in discussions about CAM.

Based on our findings, several recommendations can be drawn to strengthen cancer care in resource-limited settings:

- **At the policy level**, efforts should focus on ensuring equity in access to essential medicines, diagnostics, and qualified human resources.
- **At the health system level**, strengthening public awareness about digestive cancers is essential. Efforts should promote early detection and timely diagnosis through enhanced training of primary care providers and the establishment of clear referral pathways, and continuous access to essential medicines, diagnostic tools, and qualified health professionals.
- **At the clinical practice level**, supportive care should be strengthened, including pain and side-effect management, nutritional and psychological support, and supervising patients in their use of evidence-based complementary medicine alongside biomedical treatment. The approach should be holistic, respecting patients’ cultural values and addressing psychosocial, economic, and spiritual dimensions. It is also important to encourage patients not to ignore persistent symptoms, to talk openly with their doctors about any complementary remedies or practices they use, and to remain engaged in their care by asking questions and following treatment plans as closely as possible.
- **At the social and family level**, the central role of family and caregivers should be acknowledged, with care models integrating them as key partners in treatment adherence and emotional support.

This study has several strengths. To the best of our knowledge, it is the first qualitative study to explore diagnosis, care pathways, and the use of complementary and alternative medicine among patients with digestive cancers in Benin. Its qualitative design enabled an in-depth exploration of patients’ lived experiences, capturing their perceptions and expectations regarding cancer care and informing targeted recommendations. However, some limitations must be acknowledged. Social desirability bias may have influenced patients’ responses, although this risk was mitigated by creating a climate of trust and emphasizing the non-judgmental nature of the study. Additionally, despite reflexive practices, the researchers’ biomedical backgrounds and sociocultural positioning may have influenced both data collection and interpretation, particularly in shaping how participants framed their experiences and how themes were constructed. The study did not include healthcare providers’ perspectives, which could have provided a complementary view on the care pathways. However, this aspect is being addressed in a separate study, for which data collection is currently ongoing. A further limitation is that we did not collect detailed information on the frequency of CAM use. As participant inclusion was based on any self-reported use, individuals with potentially diverse patterns of engagement with CAM were grouped together. Although this heterogeneity was partially addressed through a qualitative typology of user profiles (pro-CAM, skeptical, and hybrid users), the absence of systematic data on usage patterns limits the depth of interpretation of our findings. Finally, as a cross-sectional study, it could not capture changes in patients’ experiences or practices over time. Despite these limitations, the study provides original insights into the lived experiences of digestive cancer patients, particularly regarding diagnostic and care pathways, and highlights the interplay between biomedical care, CAM practices, and broader social and cultural factors. Moreover, it adheres to established standards of scientific rigor in qualitative research, including achievement of thematic saturation, and reflexivity in interpretation.

## Conclusion

This study highlights the major challenges faced by patients with digestive cancers, including complex and delayed diagnostic pathways, financial barriers, and the burdens of treatment. It also shows that many patients turn to complementary and alternative medicine in search of well-being, reflecting cultural values and gaps in conventional care. These findings emphasize the need for holistic, patient-centered models that combine biomedical treatment with psychosocial, cultural, and spiritual support. To improve quality of life and clinical outcomes in resource-limited settings, it is essential to raise public awareness for early detection, expand supportive and integrative approaches, and encourage open dialogue between physicians and patients about CAM.

## Supplementary Information

Below is the link to the electronic supplementary material.

Supplementary file1 (PDF 56 KB)

Supplementary file2 (PDF 186 KB)

## Data Availability

Due to the potential for breach of confidentiality, the interview transcripts are not available.

## Declarations

### Generative AI and AI-assisted technologies in the writing process

During the preparation of this work, the authors used ChatGPT-4 (OpenAI, San Francisco, CA, USA) in order to assist with translation (French → English). After using this tool, the authors reviewed and edited the content as needed and take full responsibility for the content of the published article.
