# Supplementary material for: Diagnosis, care pathways, and Complementary and Alternative Medicine (CAM) use among digestive cancer patients in Benin: a qualitative study
Source: Support Care Cancer. 2026 Jun 9;34(7):632. doi: 10.1007/s00520-026-10853-1 (PMC13246817; doi:10.1007/s00520-026-10853-1)
Supplement: Supplementary file 1 — Supplementary file1 (PDF 56 KB) [file 520_2026_10853_MOESM1_ESM.pdf]

Interview guide

Introduction

Hello, how are you today?

We will start by introducing ourselves.

First, we would like to thank you for agreeing to take part in this study.

The aim of this interview is to better understand the circumstances surrounding your diagnosis with a digestive cancer, as well as the obstacles you may have encountered during the diagnostic or care process. We will also discuss your experience with conventional treatments and your use of complementary and alternative medicine (CAM).

Please note that your answers will remain anonymous and your personal data will be kept strictly confidential. You are free to end the interview at any time if you wish.

To facilitate the analysis, we would like to record this interview. The recording will be used only for the purposes of this study and will be deleted once the analysis is complete. Do you agree to participate and to authorize the recording of our discussion?

Part 1: interview guide questions

- **Could you tell us your story with cancer, from the first symptoms to the announcement of the diagnosis?**

Follow-up: What were the first signs or symptoms that led you to seek medical care?

- **Did you face any difficulties in accessing diagnostic tests? If yes, which ones?**

Follow-up: Do you think the diagnosis could have been made earlier?

- **In your opinion, what could help improve the early diagnosis of the disease?**

Follow-up: Are there any actions or measures you would have liked to see implemented?

- **How did you experience your medical management with conventional treatments (chemotherapy, radiotherapy, surgery, stoma, etc.)?**

Follow-up: Which aspects of the treatment seemed the most difficult for you?

- **What types of complementary or alternative medicine have you used?**
- **What motivated you to turn to these practices?**

- **What kinds of changes, if any, did you notice after using these complementary medicines or therapies?**

Follow-up: In what way did these medicines or therapies help you cope better with your illness?

- **What do you expect from healthcare professionals regarding your well-being and the integration of complementary medicine into your care?**

Are there any other elements you would like to share regarding your experience with the diagnosis or treatment of digestive cancers?

Closing the Interview

Thank you very much for your time and for sharing your experience. Your contribution is valuable for this study. Please do not hesitate to contact me if you have any questions or additional information to provide.

Part 2: Socio-demographic data

Age:

Sex:

☐ Male

☐ Female

Marital status:

☐ Single

☐ Married / In a relationship

☐ Divorced

☐ Widowed

Educational level:

☐ Illiterate

☐ Primary

☐ Secondary

☐ University

Occupation:

☐ Unemployed

☐ Retired

☐ Student

☐ Employed
