# Supplementary material for: Diagnosis, care pathways, and Complementary and Alternative Medicine (CAM) use among digestive cancer patients in Benin: a qualitative study
Source: Support Care Cancer. 2026 Jun 9;34(7):632. doi: 10.1007/s00520-026-10853-1 (PMC13246817; doi:10.1007/s00520-026-10853-1)
Supplement: Supplementary file 2 — Supplementary file2 (PDF 186 KB) [file 520_2026_10853_MOESM2_ESM.pdf]

Themes and sub-themes	Number of citations	Cited by	Text passages	Illustrative quote
**Theme 1: Early symptom appraisal and misinterpretation**				
Sub-theme: Initial minimization or trivialization of symptoms.	7	P01, P02, P06, P07, P08, P09, P10	9	**P01:** "…I thought it was just some passing stomach aches…I told myself, well, it's not really a problem as such"
Sub-theme: Lack of awareness regarding digestive cancers	3	P05, P07, P10	3	**P07:** "…so here is cancer, which wasn't something we used to hear much about in Africa. We were more familiar with diseases like malaria, hemorrhoids…. Now cancer comes along, like which make it difficult to pass stool. So, if there could be an awareness campaign so that people are informed and know the measures or precautions to take to avoid it, that would help."
**Theme 2: Navigating diagnosis within constrained health systems**				
Sub-theme: Medical wandering and diagnostic confusion	3	P01, P03, P06	15	**P03:** "…then they said there's the beginning of malaria or the beginning of a bacterial infection, that's all, it's nothing serious."
Sub-theme: Diagnosis through symptoms or emergency	10	P01, P02, P03, P04, P06, P07, P08, P09, P10, P11	22	**P09:** "I started having recurring abdominal pains."
Sub-theme: Delay in diagnosis	7	P02, P04, P05, P06, P07, P10, P11	12	**P05:** "If the illness could be diagnosed earlier, I wouldn't have had to suffer so much and I wouldn't have had to spend so much."
Sub-theme: Discomfort of diagnostic examinations	2	P03, P07	2	**P03:** I had a fibroscopy. Wow, it's disgusting."
Sub-theme: Disclosure and experience of cancer diagnosis	4	P03, P04, P05, P06	6	**P06:** At that moment, he told me it was stomach cancer, it hurt me a lot."<br>**P03:** "They told me it was cancer and all that, I said it's not a problem, you know."<br>"I wasn't traumatized by the announcement, no, not at all."
**Theme 3: Structural vulnerability shaping access to care**				
Sub-theme: Financial barriers in accessing diagnostic tests and treatments	9	P02, P03, P05, P06, P07, P08, P10	25	**P06:** "I encountered a lot of difficulties in accessing diagnostic tests, even the treatment I haven't completed because I no longer have the means."<br>"The cost is rather high, the scan is 160,000 FCFA."<br>**P08:** "I had too much debt, so finding money to continue with chemotherapy and other treatments had become impossible, impossible, impossible."
Sub-theme: Availability and accessibility of services	4	P01, P03, P07, P10	14	**P01:** "Not only are these medicines out of stock, but you also have to search far to find them."

				"There are some prescribed medicines that you simply won't find."
Sub-theme: Organizational constraints	2	P01, P10	4	**P10:** "I also think there is a shortage of staff. I wanted to have a colonoscopy in January, and they told me all the doctors were already booked, so my appointment was pushed to March."
Sub-theme: Insurance limitations	2	P04, P06	3	**P06:** "The medicines, we don't buy them with the insurance coverage. If I want to do tests, I use the coverage; for hospitalization, it's covered; for procedures, it's covered that's it. But not for buying the medicines, which are expensive"
**Theme 4: Living with cancer: biographical disruption and adaptation process**				
Sub-theme: Physical impact	4	P02, P05, P09, P10	7	**P05:** "There is also physical pain and emotional pain.
Sub-theme: Psycho-social impact	4	P02, P05, P06, P09	6	**P02:** "…it ruins you mentally."<br>**P09:** "It negatively affected the proper functioning of my family, whether it was the children who constantly saw everything I was going through, with the comings and goings, the treatments, the hospital stays, but also on the financial level."
Sub-theme: Financial impact	3	P02, P06, P09	3	**P09**<br>"It negatively affected… but also financially.
Sub-theme: Coping strategies: adaptation and resilience	4	P02, P03, P04, P09	13	**P02:** "As for me, I am strong mentally I am strong because the limits we set for ourselves are the limits of our mentality. If you want to, you can."
Sub-theme: Family support	7	P01, P02, P03, P04, P05, P07, P11	11	**P02:** "If my parents had not supported me, I am not sure I would have been able to continue until the end."
**Theme 5: Therapeutic navigation and medical pluralism**				
Sub-theme: Chemotherapy: benefits and challenges	11	P01, P03, P04, P05, P10, P09, P04, P05, P06, P07, P09, P10	22	**P04:** "The chemotherapy treatment is really beneficial."<br>**P05:** "For me, chemotherapy was difficult, very difficult. At the beginning, I had a loss of appetite, I didn't feel like eating at all, and on top of that I had a dry throat. This whole thing really tires me out (interjection)."<br>**P09:** "I noticed that chemotherapy didn't really give me satisfaction, the treatment with chemotherapy was not a success, it was a total failure."<br>**P01:** "The intensity of the sessions can be an obstacle."
Sub-theme: Discomfort and difficulties related to the stoma	3	P03, P07, P09	11	**P03:** "…ah, the stoma, wow, the stoma! waa, that was the most unpleasant thing."
Sub-theme: CAM profiles (pro, septikal, hybrid, passive)	11	P01,P02,P03,P04,P05,P06,P07,P08,P09,P10,P11	22	**P02:** "It didn't just bring me peace of mind, I would say it contributed 70% to my healing. If I had to evaluate, I'd say

				70% of my recovery came from that, compared to what conventional medicine gave me.”
Sub-theme: Types of CAM				
• Aromatherapy;	2	P03, P11	5	**P03:** “I was also doing aromatherapy, that was systematic.”
• Dietary supplements and vitamins;	3	P03, P05, P09	3	**P05:** “I also used certain vitamins and dietary supplements, but not often, you know.”
• Fasting and dietary practices;	1	P02	1	**P02:** “I did a drastic fast of 5 days without drinking or eating. After 5 days, I started drinking to eliminate toxins and all that, and it was only after the 10th day that I began to eat a bit of fruit.”
• Spirituality of connection;	3	P02, P03, P09	7	**P03:** “…spirituality is essential for me, that comes first… Because through spirituality you must know who you are, what you are capable of, what you have, and what you can achieve.”
• Religious spirituality;	11	P01, P02, P03, P04, P05, P06, P07, P08, P09, P10, P11	38	**P04:** “For complementary medicine, there are elements that can be added to conventional medicine, such as prayers.”<br>“Afterwards, I entrusted myself to God, and I noticed that things improved.”
• Cold baths;	1	P02	1	**P02:** “…the cold bath that I take, I used to buy ice cubes and put them in my bath. Apparently, that also slowed down the multiplication of those cells.”
• Chinese medicine;	1	P03	1	**P03:** Chinese medicine, there are some things in it, I don’t take everything, you know”
• Phytotherapy (herbal medecines);	9	P01, P02, P03, P04, P06, P07, P08, P10, P11	31	**P02:** “So I treated the problems with the decoctions that were suggested to me; I did it with medicinal plants.”
• Traditional healers	1	P09	2	**P09:** “I combined the treatments from traditional healers.”
Sub-theme: Motivations for CAM use				

• Due to financial difficulties<br>• Dissatisfaction with conventional treatments<br>• Worsening of clinical symptoms<br>• Better living with the illness	2<br>1<br>1<br>1	P06, P10<br>P10<br>P02<br>P11	4<br>1<br>1<br>1	**P06:** "I started by taking herbal teas. Since I no longer have the means, what am I going to do?"<br>**P10:** "I didn't find the complete satisfaction I wanted, so I turned to that."<br>**P02:** "It was when it started to get worse that I turned to alternative medicine, and I began making decoctions to see."<br>**P11:** "We can live with the illness; in any case, the illness is already there. We'll improve things that's it, especially that. If we can do it, it would be perfect."
Sub-theme: Sources of information on CAM<br>• Documentation and online search for testimonials<br>• Influence of the social circle (family, friends)	 5<br>4	 P02, P03, P04, P05, P07<br>P01, P04, P07, P10	 11<br>6	**P02:** "It's research, on the internet."<br>"I started doing research on the illness online those people who got through it, how did they go about it?"<br>**P07:** "…From relatives who give you recipes: take this, drink this." **...**"Even on social media you can find some."
Sub-theme: Perceived benefits of CAM for quality of life and coping<br>• Cultural meanings & legitimacy of CAM<br>• Importance of promoting research on CAM<br>• Integrative approach to CAM and cultural representations	 5<br>3<br>6	 P02, P05, P06, P09, P11<br>P02, P08, P09<br>P03, P07, P08, P09, P10, P11	 6<br>6<br>8	**P05:** "So all of that really helped me to live better with the illness."<br>**P02:** "This medicine needs to be supported, we need to engage in that research." ..."I think that 80% of us turn to this medicine; it must not be neglected."<br>**P09:** "Because with conventional medicine and traditional medicine, we can try to find a middle ground, to find some alignment that can still relieve patients."
**Theme 6: Expectations of holistic and integrated care**<br>• Sub-theme: Need for psycho-social support<br>• Sub-theme: Need to be supported by the physician in the use of CAM<br>• Sub-theme: Need for dietary advice	 5<br>1<br>1	 P04, P05, P07, P09, P10<br>P04<br>P04	 6<br>2<br>1	**P07:** "Psychological support would also be good."<br>**P04:** "If doctors also supported us regarding what we take or do outside of the care we receive at the hospital, that would be good, because as long as this or that practice gives me satisfaction, I'm in favor of it."<br>**P04:** "Advice on diet."

• Sub-theme: Expectations of involvement of the healthcare system in the management of care<br>• Sub-theme: The importance of establishing a relationship of trust between the physician and the patients	5<br><br><br>2	P06, P07, P08, P09, P10<br><br><br>P04, P10	7<br><br><br>4	**P06**: "It is the government that should help us and provide subsidies so that we can complete the treatment; otherwise, it is not good."<br>**P10:** "…because the way he explains things to you gives you confidence, and that alone can heal you. It would also be good if doctors were closer to the patients."
